# Hypothyroidism and related comorbidities on the risks of developing tinnitus

**DOI:** 10.1038/s41598-022-07457-0

**Published:** 2022-03-01

**Authors:** Alan Hsu, Yung-an Tsou, Tang-Chuan Wang, Wen-Dien Chang, Cheng-Li Lin, Richard S. Tyler

**Affiliations:** 1 Department of Otolaryngology-Head and Neck Surgery, China Medical University Hsinchu Hospital, Zhubei City, Hsinchu County Taiwan; 2 School of Medicine, College of Medicine, China Medical University, Taichung, Taiwan; 3 Department of Public Health, College of Public Health, China Medical University, Taichung, Taiwan; 4 Department of Sport Performance, National Taiwan University of Sport, Taichung, Taiwan; 5 Management Office for Health Data (DryLab), Clinical Trial Center (CTC), China Medical University Hospital, Taichung, Taiwan; 6 Department of Otolaryngology-Head and Neck Surgery, University of Iowa, Iowa City, IA USA

**Keywords:** Neuroscience, Physiology

## Abstract

This is a retrospective longitudinal study that uses data from the National Health Insurance Research Database (NHIRD) of Taiwan of which hypothyroid patients who received a diagnosis between 2000 and 2010 were selected and followed up until 2011. The primary outcome of this study was the occurrence of tinnitus (ICD-9-CM code 388.3). The relevant comorbidities were selected as potential confounders according to the literature, which included vertigo (ICD-9-CM code 386), insomnia (ICD-9-CM code 780), anxiety (ICD-9-CM code 300.00), and hearing loss (ICD-9-CM code 388–389). The overall incidence of tinnitus was significantly higher in the hypothyroidism cohort than in the non-hypothyroidism cohort (9.49 vs. 6.03 per 1000 person-years), with an adjusted HR of 1.35 (95% CI 1.18–1.54) after adjusting potential confounders. The incidences of tinnitus, as stratified by gender, age, comorbidity, and follow-up time, were all significantly higher in the hypothyroidism cohort than those in the non-hypothyroidism cohort. The incidence of tinnitus significantly increased with age (aHR = 1.01, 95% CI 1.01–1.02). In conclusion, we report the relationship between hypothyroidism and the increased risk for tinnitus. We also found that hypothyroidism patients are at increased risk of developing tinnitus when associated with comorbidities including vertigo, hearing loss, and insomnia.

## Introduction

Tinnitus is a very common symptom seen among the general populace^1^ with the most common cause of tinnitus being hearing loss^1^. Unfortunately, there currently does not exist an universal consensus on the definition for tinnitus and so reported prevalence in population studies has varied from 5.1 to 42.7%^2^.

Despite its prevalence, there is no general consensus on the mechanism that would adequately explain how altered thyroid hormone levels may lead to tinnitus. However, it is known that the thyroid hormones are known to be contributors to many developmental processes of the body, including the maturation of the cochlea^3^. Therefore, any dysfunction of the thyroid can have a knock-on effect on the maturation of the auditory system including the organ of corti^4^, of which one if its many manifestations could be tinnitus.

In recent years, other mechanisms have been proposed which includes the effect on the interplay between adrenaline receptors and the thyroid hormones^5^. One of the ways the thyroid gland regulates the sympathetic system is via altering the spread and functionality of the adrenergic receptors from the tissues of the body. Any changes to the normal thyroid status will therefore affect this balance and ultimately, affect the overall physiological function of the body^6^. In addition, the peripheral blood flow is also known to be under the control of the thyroid hormones as well. The mechanism for this is not very well understood, but may involve modulation of the potassium channels causing changes in the gradient of the Na^+^/K^+^ ions across the vascular walls. Another way in which the thyroid controls the blood flow is through changes in the levels of vasoactive hormones released by the vascular endothelium^7^.

Another point to make is that one of the many regulations of the cochlear blood supply includes the sympathoadrenal system. Sympathetic nerve fibers running from the stellate ganglia and superior cervical ganglia has been shown to terminate on the spiral modiolar artery. This innervation is partly responsible in how the cochlear vasculature maintains its vascular tone. To put it simply, the flow of blood in any vessel is regulated by the diameter of the vascular channel. Activation of the sympathetic nerves will contract the smooth muscle cells of the vasculature. Consequently, the reduction of the diameter of the blood vessels will result in the reduction of cochlear blood flow. Conversely, deactivation results in an increase in cochlear blood flow^8^. Further animal studies have confirmed these theories. Spoendlin et al. was the first to demonstrate the presence of sympathetic nerve fibers regulating cochlear blood flow^9^. Hozawa et al. used immunohistochemical studies on monkeys to further demonstrate the presence of sympathetic related neurotransmitters in the inner ear^10^. Gil-Loyzaga et al. also contributed by showing that cervical ganglionectomy in rat models will result in noticeable decrease in sympathetic neurotransmitters to be found in the rat cochlea^11^. Finally, Wangemann et al. demonstrated in electrophysiological animal studies on guinea pigs that the diameter of the vascular vessels to the cochlea can also be manipulated through the stimulation of the sympathetic nerves^8^.

Therefore, taken together, it can be suggested that cochlear pathologies could be a consequence from any factors that would cause sympathetic imbalance; such as, altered homeostasis of the thyroid hormones. And finally, the manifestation of this hypoxic insult to the cochlear may clinically present as tinnitus seen among patients with hypothyroidism.

Tinnitus is also known to be a clinical condition with multiple comorbidities and has been known to be associated with other non-ear related diseases such as atherosclerosis, diabetes and thyroid disorders^12^. Links between tinnitus and hypothyroidism has been described by numerous authors but these are limited by their small sample sizes. Anand et al.^13^ reported tinnitus in 3 patients (15%) with hypothyroidism. Santosh et al.^14^ evaluated 35 patients with Meniere’s disease like symptoms including tinnitus and 12 patients (34%) were associated with hypothyroidism. Santos et al. found^15^ 5 hypothyroid patients (16.67%) reported tinnitus. Singh et al.^16^ reported 13 hypothyroid patients (26%) that complained of tinnitus.

Extensive epidemiological studies allow us to use a large data set to investigate the prevalence of specific clinical conditions and explore their relationship with predesignated variables. These studies and its findings can contribute to a more comprehensive knowledge for risk factors for a certain condition and also assist in the clinical management of a specific patient cohort. However, no extensive studies to date have been done to look into the association between tinnitus and hypothyroidism. Our present study is the first to use real-world data to explore the risks of developing tinnitus among a large population-based cohort of hypothyroidism patients. We also seek to estimate the relationship between the chances of developing tinnitus and other covariables such as thyroid status, age, gender, vertigo, insomnia, anxiety and hearing loss.

## Material and methods

This population-based retrospective cohort study was designed to investigate the risk of tinnitus between populations with and without hypothyroidism using claims data of the Taiwan National Health Insurance Research Database (NHIRD). Since its establishment in 1995, Taiwan's National Health Insurance Program has provided universal and comprehensive health care for approximately 99% of Taiwan residents to date. NHIRD was released by the National Health Research Institute (NHRI) for research purposes and contains detailed medical records, including demographic data, outpatient visits and hospitalizations, diagnoses, procedures, surgeries, and prescription details, as described previously. The data utilized in this study was the Longitudinal Health Insurance Database (LHID 2000), a representative subset of the NHIRD. The LHID 2000 consists of 1 million randomly collected samples. According to an NHRI report, there is no difference in age or sex distribution between the populations of the LHID and NHIRD. The identification of all insured people in the database was encrypted to protect personal privacy, and informed consent was waived. Diseases were classified based on the International Classification of Diseases, Ninth Revision, Clinical Modification (ICD-9-CM). The accuracy and validity of NHIRD diagnosis codes have been documented.

### Study population

From LHID 2000, the case cohort consisted of patients with newly diagnosed hypothyroidism (ICD-9-CM code 244) from January 1, 2000, to December 31, 2010. The index date of the hypothyroidism cohort was fixed as the initial hypothyroidism diagnosis date. The comparison cohort were individuals without hypothyroidism diagnoses selected from the same database and randomly frequency matched by age (with the span of every 5 years), sex, and the year of the index date at a 1:4 ratio. Those in the comparison cohort were randomly assigned the same index date as the matched cases. Both cohorts excluded individuals with a history of tinnitus before the index date.

### Outcome and comorbidity

The primary outcome of this study was defined as the occurrence of tinnitus (ICD-9-CM code 388.3). The relevant comorbidities were selected as potential confounders according to the literature, which included vertigo (ICD-9-CM code 386), insomnia (ICD-9-CM code 780), anxiety (ICD-9-CM code 300.00), and hearing loss (ICD-9-CM code 388-389). The comorbidities identified according to their diagnoses before the index date. The urbanization level was categorized by the residential area's population density into four levels, with level 1 as the most urbanized and level 4 as the least urbanized. All subjects were followed from the index date until the tinnitus occurred, withdrew from the NHI system, death, or December 31, 2011, whichever came first.

### Statistical analysis

Differences between the hypothyroidism cohort and the non-hypothyroidism cohort in terms of the descriptive data, including age, gender, urbanization level, and comorbidity, were tested by t test and Chi-square. Continuous data were presented as means (standard deviation), and categorical data were presented as numbers (percentages). Incidence rate (IR) was defined as the number of events divided by the sum of follow-up time per 1000 person-years. To determine the risk of tinnitus between the patients with and without hypothyroidism, Cox proportional hazards regression models were used to estimate crude and adjusted hazard ratios (HRs) and 95% confidence intervals (CIs). Besides, stratified analyses based on gender, age (≤ 39, 40–54, ≥ 55), comorbidity, and follow-up time were performed to examine the consistency of hypothyroidism's relationship on the risk of tinnitus. We further evaluated the interaction effect of hypothyroidism and comorbidities on the risk of tinnitus. The cumulative incidence of tinnitus for patients with and without hypothyroidism was estimated using the Kaplan–Meier method, with curve differences tested by log-rank tests. All data processing and statistical analyses were performed using SAS version 9.4 software (SAS Institute, Cary, NC, USA). The threshold for statistical significance was set at *p* < 0.05.

### Ethics approval

This study was conducted strictly according to the guidelines and regulations of the Declaration of Helsinki^17^ and the protocols for this study was approved by the China Medical University Hospital Research Ethics Committee at the China Medical University Hospital (CMUH104-REC2-115(CR-5)). As the Taiwan National Health Insurance dataset is comprised of already de-identified data for research usage, the requirement of written consent from study participants were deemed unnecessary and waived by the Ethics Research Committee.

## Results

Table 1 reveals the distributions of gender, age, urbanization level, and comorbidities between the hypothyroidism and non-hypothyroidism cohorts. The study population included 6062 cases and 24,248 matched controls, with well-balanced distributions of gender and age. The mean age was approximately 49 years old, and 81.6% of the patients were female. Patients in the hypothyroidism cohort had a significantly higher prevalence of vertigo, insomnia, anxiety, and hearing loss than the non-hypothyroidism cohorts (all *p* < 0.05).

**Table 1:** Comparisons in demographic characteristics and comorbidities in patient with and without Hypothyroidism.

	Hypothyroidism		*p* value
	No (N = 24,248)	Yes (N = 6062)	
**Gender**			0.99
Women	19,784 (81.6)	4946 (81.6)	
Men	4464 (18.4)	1116 (18.4)	
**Age stratified**			0.99
≤ 39	7527 (31.0)	1882 (31.0)	
40–54	7993 (33.0)	1998 (33.0)	
≥ 55	8728 (36.0)	2182 (36.0)	
Age, mean ± SD ^a^	49.5 (16.7)	49.9 (16.5)	0.10
**Urbanization level**			< 0.001
1 (highest)	7421 (30.6)	2020 (33.3)	
2	7075 (29.2)	1751 (28.9)	
3	4284 (17.7)	977 (16.1)	
4 (lowest)	5468 (22.6)	1314 (21.7)	
**Comorbidity**			
Vertigo	1746 (7.20)	717 (11.8)	< 0.001
Insomnia	9608 (39.6)	3340 (55.1)	< 0.001
Anxiety	2101 (8.66)	1063 (17.5)	< 0.001
Hearing loss	271 (1.12)	108 (1.78)	< 0.001

Chi-Square Test, ^a^t-test.

^†^The urbanization level was categorized by the population density of the residential area into 4 levels, with level 1 as the most urbanized and level 4 as the least urbanized.

Table 2 reveals the incidence of tinnitus of the study groups. At the end of the study, the mean follow-up time was 5.34 (SD = 3.47) years and 5.45 (SD = 3.46) years for hypothyroidism non-hypothyroidism cohorts, respectively. The overall incidence of tinnitus was significantly higher in the hypothyroidism cohort than in the non-hypothyroidism cohort (9.49 vs. 6.03 per 1000 person-years), with an adjusted HR of 1.35 (95% CI 1.18–1.54) after adjusting potential confounders. The incidences of tinnitus, as stratified by gender, age, comorbidity, and follow-up time, were all significantly higher in the hypothyroidism cohort than those in the non-hypothyroidism cohort.

**Table 2:** Comparison of incidence densities of Tinnitus and hazard ratio between with and without Hypothyroidism by demographic characteristics and comorbidity.

	Hypothyroidism						Crude HR*(95% CI)	Adjusted HR^†^ (95% CI)
	No			Yes				
	Event	PY	Rate^#^	Event	PY	Rate^#^		
All	797	132,251	6.03	307	32,365	9.49	1.57 (1.38, 1.80)***	1.35 (1.18, 1.54)***
**Gender**								
Women	688	110,986	6.20	260	27,280	9.53	1.54 (1.33, 1.77)***	1.32 (1.14, 1.52)***
Men	109	21,265	5.13	47	5086	9.24	1.80 (1.28, 2.53)***	1.56 (1.09, 2.21)*
**Stratify age**								
≤ 39	148	45,049	3.29	67	11,243	5.96	1.82 (1.36, 2.42)***	1.48 (1.10, 1.99)**
40–54	288	46,104	6.25	115	11,361	10.1	1.62 (1.31, 2.01)***	1.38 (1.11, 1.73)**
≥ 55	361	41,098	8.78	125	9761	12.8	1.46 (1.19, 1.79)***	1.24 (1.01, 1.52)*
**Comorbidity**^‡^								
No	313	83,895	3.73	97	14,834	6.54	1.74 (1.39, 2.18)***	1.78 (1.41, 2.23)***
Yes	484	48,356	10.0	210	17,532	12.0	1.20 (1.02, 1.41)*	1.23 (1.05, 1.45)*
**Follow-up time (years)**^‡^								
≤ 5	533	62,200	8.57	203	15,115	13.4	1.52 (1.30, 1.79)***	1.30 (1.10, 1.53)**
> 5	264	41,784	6.32	104	10,120	10.3	1.63 (1.30, 2.04)***	1.51 (1.19, 1.90)***

Rate^#^, incidence rate, per 1000 person-years; Crude HR*, relative hazard ratio; Adjusted HR^†^: multivariable analysis including age, and comorbidities of vertigo, insomnia, anxiety, and hearing loss; *p < 0.05, **p < 0.01, ***p < 0.001.

Comorbidity^‡^: Patients with any one of the comorbidities vertigo, insomnia, anxiety, and hearing loss were classified as the comorbidity group.

^‡^The follow-up time is partitioned into 2 segments (years ≤ 5, and > 5 years) by median.

Tinnitus associated with hypothyroidism and other covariates were reported in Table 3. The incidence of tinnitus significantly increased with age (aHR = 1.01, 95% CI 1.01–1.02). In addition, patients with vertigo (aHR = 1.63, 95% CI 1.37–1.93), insomnia (aHR = 1.66, 95% CI 1.46–1.90), anxiety (aHR = 1.43, 95% CI 1.21–1.70), and hearing loss (aHR = 4.01, 95% CI 3.09–5.21) had a significantly higher risk of tinnitus than that of patients without these comorbidities.

**Table 3:** Cox model with hazard ratios and 95% confidence intervals of Tinnitus associated with Hypothyroidism and covariates.

Variable	Crude		Adjusted^†^	
	HR	(95% CI)	HR	(95% CI)
**Gender**				
Women	1.16	(0.98, 1.37)	–	–
Men	1	(reference)	–	–
Age, years	1.02	(1.02, 1.03)***	1.01	(1.01, 1.02)***
**Urbanization level**				
1 (highest)	1	(reference)	–	–
2	1.01	(0.86, 1.17)	–	–
3	1.00	(0.83, 1.19)	–	–
4 (lowest)	1.09	(0.93, 1.28)	–	–
**Baseline comorbidities (yes vs no)**				
Hyperthyroidism	1.57	(1.38, 1.80)***	1.35	(1.18, 1.54)***
Vertigo	2.75	(2.35, 3.23)***	1.63	(1.37, 1.93)***
Insomnia	2.27	(2.01, 2.56)***	1.66	(1.46, 1.90)***
Anxiety	2.25	(1.91, 2.64)***	1.43	(1.21, 1.70)***
Hearing loss	6.22	(4.81, 8.04)***	4.01	(3.09, 5.21)***

Crude HR*, relative hazard ratio; adjusted HR^†^: multivariable analysis including age, and comorbidities of vertigo, insomnia, anxiety, and hearing loss; *p < 0.05, **p < 0.01, ***p < 0.001.

**Figure 1:**
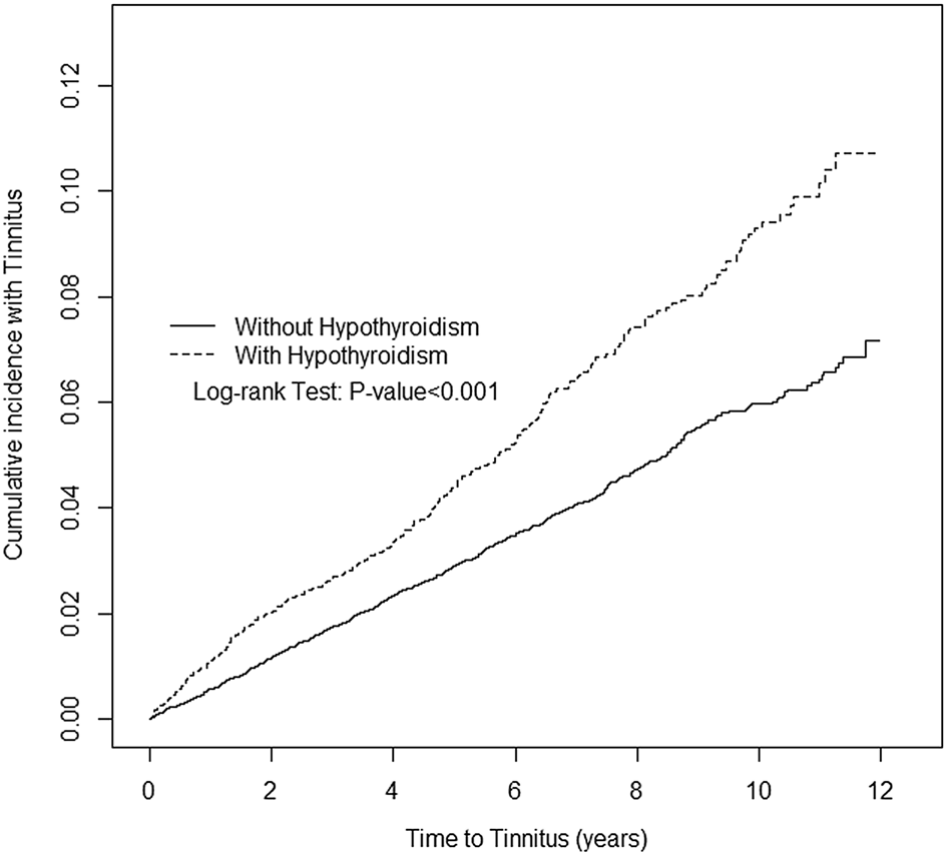
Cumulative incidence of Tinnitus for patients with (dashed line) or without (solid line) Hypothyroidism.

Results are shown in Table 4, demonstrating an interaction effect between hypothyroidism and comorbidities (including vertigo, insomnia, and anxiety) on the risk of tinnitus. Compared to non-hypothyroidism patients without vertigo, patient with both hypothyroidism and vertigo exhibited the highest risk of tinnitus (aHR = 2.72, 95% CI 2.08–3.55), followed by non-hypothyroidism patients with vertigo (aHR = 2.33, 95% CI 1.90–2.84) and hypothyroidism patients without vertigo (aHR = 1.59, 95% CI 1.37–1.84). Compared to non-hypothyroidism patients without insomnia, patients with both hypothyroidism and insomnia exhibited the highest risk of tinnitus (aHR = 2.61, 95% CI 2.18–3.11), followed by non-hypothyroidism patients with insomnia (aHR = 2.04, 95% CI 1.77–2.36) and hypothyroidism patients without insomnia (aHR = 1.73, 95% CI 1.41–2.14). Compared to non-hypothyroidism patients without anxiety, non-hypothyroidism patients with anxiety exhibited the highest risk of tinnitus (aHR = 2.21, 95% CI 1.81–2.70), followed by a patient with both hypothyroidism and anxiety (aHR = 2.12, 95% CI 1.63–2.75) and hypothyroidism patients without anxiety (aHR = 1.64, 95% CI 1.42–1.90). Kaplan–Meier analysis illustrates that the cumulative incidence of tinnitus was significantly higher in the hypothyroidism cohort than in the non-hypothyroidism cohort (log-rank test, *p* < 0.001) (Fig. 1).

**Table 4:** Cox proportional hazard regression analysis for the risk of tinnitus-associated hypothyroidism with interaction of comorbidity.

Variables		Event	PY	Rate^#^	Adjusted HR^†^ (95% CI)	p value^§^
Hypothyroidism	Vertigo					0.05
No	No	677	124,667	5.43	1 (Reference)	
No	Yes	120	7584	15.8	2.33 (1.90, 2.84)***	
Yes	No	248	29,046	8.54	1.59 (1.37, 1.84)***	
Yes	Yes	59	3319	17.8	2.72 (2.08, 3.55)***	
Hypothyroidism	Insomnia					< 0.001
No	No	364	87,724	4.15	1 (Reference)	
No	Yes	433	44,527	9.72	2.04 (1.77, 2.36)***	
Yes	No	118	16,462	7.17	1.73 (1.41, 2.14)***	
Yes	Yes	189	15,903	11.9	2.61 (2.18, 3.11)***	
Hypothyroidism	Anxiety					< 0.001
No	No	681	123,994	5.49	1 (Reference)	
No	Yes	116	8257	14.1	2.21 (1.81, 2.70)***	
Yes	No	246	27,556	8.93	1.64 (1.42, 1.90)***	
Yes	Yes	61	4810	12.7	2.12 (1.63, 2.75)***	
Hypothyroidism	Hearing loss					0.63
No	No	750	131,113	5.72	1 (Reference)	
No	Yes	47	1139	41.3	5.74 (4.25, 7.74)***	
Yes	No	292	31,930	9.14	1.60 (1.40, 1.84)***	
Yes	Yes	15	435	34.5	5.24 (3.14, 8.75)***	

Adjusted HR^†^: adjusted for age and gender. *p < 0.05, **p < 0.01, ***p < 0.001.

^§^p value for interaction.

## Discussion

This current study represents the largest sample size to date that seeks to investigate the risk of tinnitus among patients with hypothyroidism. This retrospective cohort study observed that hypothyroidism patients were more predisposed to develop tinnitus than those without hypothyroidism, even after adjusting for age, gender, economic, and hearing-related clinical comorbidities. This study was also among the largest to observe an increased risk of tinnitus among hypothyroid patients when associated with hearing loss, vertigo, and insomnia.

Other smaller studies^13,14,16^ have primarily drawn similar conclusions but are often limited by their small study size compared to ours. A prospective study by Anand et al. reported that 3 of the 20 patients with hypothyroidism had tinnitus^13^. Another one found that out of 35 patients with Meniere’s disease-like symptoms such as tinnitus surveyed, 12 of them had hypothyroidism^14^. Dos Santos et al. also revealed that 5 out of their 30 hypothyroid patients reported tinnitus^14^. Other minor studies^18–20^ also showed tinnitus being observed among small groups of hypothyroid patients, and another reported similar findings for 6 out of 30 hypothyroid patients. Intriguingly, this study by Singh et al. conducted on 50 hypothyroid patients found 13 patients having tinnitus symptoms. Singh et al. also demonstrated that out of the 13 hypothyroid patients with tinnitus, 8 of their tinnitus symptoms resolved after thyroxine treatment was initiated^16^. Malik et al.^21^ also reported reversibility of tinnitus after the treatment of hypothyroidism among its patients.

This study also showed as a secondary outcome that patients with hypothyroidism seem to have an interactional effect with comorbidities including hearing loss (aHR = 4.01, 95% CI 3.09–5.21), vertigo (aHR = 2.72, 95%, CI 2.08–3.55) and insomnia (aHR = 2.61, 95% CI 2.18–3.11) towards the risk of developing tinnitus. This study also additionally demonstrated that the most significant interactional effect between non-hypothyroidism and anxiety (aHR = 2.21, 95% CI 1.81–2.70) towards the risk of tinnitus. To date, this is the most extensive and most likely the only study where the risk factors in developing tinnitus among hypothyroid patients have been looked into. However, individual links between tinnitus and each of these comorbidities among the general population are well established among papers. In a large-scale national health survey of 21 million tinnitus sufferers, 26.9% of them (5.59 ± 0.31 million adults) reported problems with anxiety^22^. Sleep disturbance has also been reported to be related to tinnitus, and a dose–response relationship between lack of sleep and the severity of tinnitus has been found^23,24^. A cross-sectional study by Bhatia et al. on 72 hypothyroid patients found 23 patients developed vertigo and five patients developed tinnitus^15^. This demonstrates a relationship between hypothyroidism and vertigo and tinnitus and how all these interrelations remain unexplored. Finally, an association between tinnitus and hearing loss has also been a well-accepted concept. Sindusake et al. reported a modest association between hearing loss and tinnitus^25^. Phoon et al. also found that—after accounting for confounding variables like age and gender, tinnitus was again more commonly seen among those with hearing loss^26^. In their 33,168-patients study, they also reported the hearing loss as the most significant variable associated with tinnitus. In that same study, higher frequency hearing loss was found to be more correlated with tinnitus.

This study also linked the risk of developing tinnitus to an increase in age (aHR = 1.01, 95% CI 1.01–1.02). This correlates with a large epidemiological study done in Korea among 21,893 tinnitus patients, which showed that tinnitus prevalence significantly increased with age as well^27^. The median age of that study among those who developed tinnitus was 57.23 years old, while ours was 49 years old. Many aspects of the brain, such as functionality and physical anatomy changes with the aging process^28^; thus, the pathophysiology underpinning age-related decline could potentially be related to the generation of tinnitus as well^29^.

The exact cause of the association between hypothyroidism and tinnitus is still up for debate. Other mechanisms also include the previously mentioned effect the thyroid status has on sympathoadrenal system and altered cochlear blood flow. The thyroid hormone is also known to play other roles, including the nurturing and maturation of the central nervous system^30–33^. Uziel et al.^34^ hypothesized that any deficiencies in the thyroid hormone levels could also impact the otological developments, including the organ of Corti. Therefore, in theory, such deficiencies in ontological developments may have some role in the future susceptibility to manifesting tinnitus. Several animal studies have reported inner and middle ear anomalies to be associated with congenital hypothyroidism. These animal studies are usually based on mice models as the various sequence of auditory structural and functional developments are not dissimilar to the process seen among human beings^35^. In humans, changes in hearing function have been documented where there is a drop in thyroid hormone levels during sensitive stages of the neonatal and infantile periods of development^36^. Among rodents, thyroid hormone deficiencies that occur before the onset of hearing have been shown to cause irreversible changes to both the central and peripheral auditory systems^37^. The mechanisms of such changes have been hypothesized to be related to some form of changes occurring on the genetic level. Several candidate genes have been identified that potentially respond to changes in the thyroid hormone levels^35^ but the exact molecular changes underpinning their hypothyroid-induced audiological sequelae remains unclear.

This study has several strengths, which includes the use of an extensive national database based on real-world data as well as its patient privacy protection. Since this study is based on a national database; with heterogenous patients from different age groups, varying comorbidities and socioeconomic status, these data from real-world settings can further contribute to the validity of this study.

Nevertheless, a few limitations in this study deserves some discussion. Firstly, one of the most obvious limitations is the lack of a universally established clinical definition for tinnitus. This introduces a level of uncertainty about the comparability of this study with others. Furthermore, we cannot comment on the severity, and the clinical phenotypes of tinnitus encountered in our study population nor provide any figures to quantify the hearing loss noted in our data. Interestingly, among those with hearing loss suffering from tinnitus, approximately half of them reported relief from their tinnitus in one study^37^ after the implementation of hearing aids. Another study also showed improvement in tinnitus among patients with hearing loss after receiving a cochlear implant^38^. We did not explore this therapeutic relationship in our study. These, however, form interesting avenues that deserve some attention in future studies.

Our reference database also limited us to provide for other confounders like patients’ ethnicity and other specific otologic features or neurological findings. Secondly, this study was conducted in a predominately Taiwanese-Mandarin-speaking environment. However, the majority of tinnitus-related measures to date have been originally derived from English. This means that these clinical questionnaires have to be translated into Mandarin for the patients assessed in this study. This raises interesting questions about clinical tools’ reliability and validity across different cultures and languages. Unfortunately, the Taiwan National Health Insurance Database does not provide any information about the translational processes that the various hospitals in Taiwan included in this study went through and what vigorous methods of validation were adopted to ensure that the validity and reliability of these tinnitus questionnaires remained intact. Some have argued that after the translation process, the original content and its meaning that were specific to the original creator’s language would be lost. Therefore any newly translated outcome measures would lose its equivalence as a clinical surveying tool^39^. However, this limitation would be challenging to overcome as there still lacks a widely accepted methodology for translating clinical measurements^40^. Secondly, this study is limited by the type of data captured in the national insurance database. This introduces a level of uncertainty and variability when making comparisons with our data. One major point is that we cannot account for what exact type of tinnitus measurement tools used to assess the tinnitus patients in our database clinical criteria^41^.

Thirdly, this study was also limited by its retrospective nature. Therefore, it lacks any prospective data to follow up the progression or recovery in the tinnitus status among the patients with hypothyroidism. Lastly, this study's results may not be entirely comparable for other racial/ethnic groups as the original population was drawn from a predominately Taiwanese Asian demographic. A large-scale cross-sectional study from the United States has shown that tinnitus prevalence is significantly lower among Asians than other ethnic groups^42^. Ethnicity status has been suggested to be another independent factor that influences the risk of tinnitus. This confounding variable should be kept in mind when reviewing similar studies such as ours. In future, we can combine our dataset with ones from other countries that is also investigating tinnitus. This combination would introduce more diversity in terms of epidemiology and also further increase the validity of any future findings derived.

### Clinical implications

The subjective nature of tinnitus, its multitude of risk factors, and various clinical phenotypes contribute to its challenges and elusiveness. Therefore, better understanding of the various triggers and comorbid conditions (such as hypothyroidism) that may contribute to tinnitus can help better guide future healthcare professionals in their treatment and diagnostic approach. Considerable efforts have been made to provide strategies for managing suspected tinnitus cases. These strategies include guidelines and algorithms issued from relevant national medical bodies such as the United States^43^ and Germany^44^; as well as from dedicated international organizations such as the Tinnitus Research Initiative^45^. Across these guidelines, some forms of consensus do exist. Common grounds include the need to do diagnostic assessments for other comorbidities co-existing with tinnitus and as well as devising treatment plan for them when found. It is evident from this that the current prevailing model of treatment for tinnitus is to view and treat it as a symptom of consequence from several different comorbidities. The level of agreement is high among the various existing guidelines with regards to the existence and management of comorbidities such as depression, anxiety, insomnia and hearing loss among tinnitus patients^46^. Unfortunately, little attention so far has been paid to the diagnosis and management for comorbid conditions related specifically to hypothyroidism. The omission of this comorbidity of endocrine origin from standard guidelines is interesting as several authors from various studies^13–16^ have already drawn associations between these two conditions in the past. Therefore, we feel it is important to form a large study like ours to draw further associations between the conditions of tinnitus and hypothyroidism with good quality data. The paucity of high-level evidence impedes the formation of any recommendations from guidelines and therefore makes it very difficult for healthcare providers to decide what is best in terms of the assessments and treatment options for such a subgroup of tinnitus patients. It is entirely possible to theorize that the early management of comorbidities of tinnitus; such as hypothyroidism in this case, could have a therapeutic effect including preventive or at least earlier recovery from tinnitus as well. This therapeutic relationship has been hinted by Malik et al.^21^ and Singh et al.^16^, where they reported resolution of tinnitus symptoms among patients after respective treatment of their hypothyroid status. Further rigorous studies with different study designs, larger sample sizes and combination with other national database with more ethnic diversity however are still warranted to verify findings and better understand tinnitus.

## Conclusion

To the best of our knowledge, this is the first study to use a large population base to investigate the risk of developing tinnitus among patients with hypothyroidism and the various factors that may contribute to its development. In conclusion, we report the relationship between hypothyroidism status and its increased risk for tinnitus based on claims data of the Taiwan National Health Insurance Research Database. We also found that those patients with hypothyroidism are at increased risk of developing tinnitus when associated with comorbidities, including vertigo, hearing loss, and insomnia.
